# Pickleball and Ball Sports-Related Ocular Trauma in the United States

**DOI:** 10.1038/s41433-025-04192-4

**Published:** 2026-01-16

**Authors:** Jainam Shah, Sachin Pathuri, Anurag Shrivastava, Joshua Ong, Jason Zheng, Ibrahim Furkan Acar, James Plotnik, Karl Golnik, Mark I. Salevitz, Alex Suh, Andrew G. Lee

**Affiliations:** 1 Albert Einstein College of Medicine, Bronx, NY USA; 2 Department of Ophthalmology and Visual Sciences, Montefiore Medical Center, Albert Einstein College of Medicine, Bronx, NY USA; 3 Department of Ophthalmology and Visual Sciences, University of Michigan Kellogg Eye Center, Ann Arbor, Michigan USA; 4 California University of Science and Medicine, Colton, CA USA; 5 University of California, Los Angeles, CA USA; 6 Division of Ophthalmology, Phoenix Children’s Hospital, Phoenix, AZ USA; 7 Department of Neurology, Barrow Neurological Institute, Phoenix, AZ USA; 8 Department of Ophthalmology, USC Roski Eye Institute, Keck School of Medicine, University of Southern California, Los Angeles, CA USA; 9 Center for Space Medicine, Baylor College of Medicine, Houston, TX USA; 10 Department of Ophthalmology, Blanton Eye Institute, Houston Methodist Hospital, Houston, TX USA; 11 The Houston Methodist Research Institute, Houston Methodist Hospital, Houston, TX USA; 12 Departments of Ophthalmology, Neurology, and Neurosurgery, Weill Cornell Medicine, New York, NY USA; 13 Department of Ophthalmology, University of Texas Medical Branch, Galveston, TX USA; 14 University of Texas MD Anderson Cancer Center, Houston, TX USA; 15 Texas A&M College of Medicine, Bryan, TX USA; 16 Department of Ophthalmology, The University of Iowa Hospitals and Clinics, Iowa City, IA USA

**Keywords:** Epidemiology, Health care

## Abstract

**Objective:**

To characterize national patterns of ocular trauma related to pickleball, dodgeball, and kickball in the United States, with emphasis on incidence, demographics, injury mechanisms, and diagnoses.

**Methods:**

Data from the U.S. Consumer Product Safety Commission’s National Electronic Injury Surveillance System (2014–2023) were reviewed. Injury cases were identified using body part codes for the eye, face, and head combined with product code 3235 (“Other Ball Sports”). Trauma narratives were subsequently manually reviewed to confirm sport involvement alongside ocular and/or orbital injury. National injury estimates were calculated using the NEISS-provided sample weights. Annual incidence per million people was calculated using data from the U.S. Census Bureau and American Community Survey. Statistical analyses included descriptive statistics and inferential testing using chi-square tests, ANOVA, and linear regression.

**Results:**

A total of 120 confirmed cases were identified, corresponding to an estimated 7974 ocular injuries (95% CI: 7184–8764). Weighted estimates attributed 3874 cases (95% CI: 3429–4319) to dodgeball, 2573 (95% CI: 2218–2928) to pickleball, and 1527 (95% CI: 1255–1799) to kickball. Pickleball injuries primarily affected older adults (mean age 58.27 years), whereas dodgeball and kickball injuries occurred mostly in youth (mean ages 13.32 and 14.88 years). Direct ball impact was the leading mechanism, with corneal abrasion as the most frequent diagnosis. Falls (15.38%) and orbital fractures (11.54%) were more common with pickleball. Linear regression demonstrated a significant increase in pickleball-related ocular trauma from 2014 to 2023 (*P* = 0.04).

**Conclusions:**

Pickleball-related ocular trauma is increasing nationally, particularly among older adults. These largely preventable injuries highlight the need for sport-specific preventive strategies and increased access to protective eyewear.

## Introduction

Ocular trauma is the leading cause of non-congenital unilateral blindness among individuals under the age of 20 in the United States (U.S.), and it has been shown to be a cause of long-term visual impairment, emotional distress, and decreased quality of life [1, 2]. Ocular injuries are of significant concern to the health system; an estimated 4.3 million of the 12 million U.S. emergency department (ED) visits for ocular conditions between 2006 and 2011 were due to ocular trauma [3]. A prior study estimated that annual hospital charges for eye injuries are approximately between $175 and $200 million [4].

Given these implications, it is crucial that the causes of ocular injuries be identified and prevented. While most sports literature focuses on musculoskeletal injuries, participation in athletics also carries a risk of ocular trauma, accounting for nearly 30,000 ED visits annually in the U.S., according to the Center for Disease Control and Prevention (CDC) [5, 6]. High-speed equipment sports such as basketball, soccer, and tennis present a high-risk of ocular injury. However, other recreational sports such as kickball and dodgeball, which carry a similar risk of eye injury due to their high-velocity projectiles and physical contact, remain understudied in the context of ocular trauma, despite their popularity.

Pickleball, one of the fastest-growing racquet sports, is increasingly recognized as a source of ocular trauma because it involves a plastic ball travelling at speeds of approximately 40 miles per hour, which increases the likelihood of sustaining high-velocity ocular injuries. The Sports and Fitness Industry Association (SFIA) reported that pickleball participation increased by 158.6% from 2020 to 2023, which is consistent with increases in ED visits for pickleball-related ocular injuries [7, 8]. Current evidence is largely provided by case series documenting complications such as corneal abrasions, retinal detachments, and hyphema. Comprehensive population-level analyses remain highly limited, constraining the ability to inform prevention strategies and fully characterise at-risk populations [7, 9].

Using data from the National Electronic Injury Surveillance System (NEISS), this study characterizes ocular trauma from pickleball, dodgeball, and kickball. Dodgeball and kickball were selected as a point of comparison to pickleball because they also involve high-velocity ball collisions, close player proximity, and widespread game play in many settings. Therefore, they provide a benchmark to determine how pickleball-related injuries may differ in terms of demographic risk, mechanisms of injury, and clinical presentations. We describe national patterns in demographics, mechanisms of trauma, diagnoses, and seasonal trends to identify at-risk groups, common settings, and injury types. These findings aim to guide acute care, targeted prevention, and public health planning. Given pickleball’s rapid growth and the absence of comprehensive population-level data on its ocular complications, this study provides foundational epidemiologic insight essential for reducing preventable vision loss across a diverse athletic population.

## Methods

### NEISS search strategy

A retrospective cohort study was conducted using the NEISS, a publicly available injury surveillance database maintained by the U.S. Consumer Product Safety Commission (CPSC). The NEISS collects data from a stratified sample of approximately 100 hospitals representing over 5000 emergency departments (EDs) across the U.S. Each injury case receives a statistical weight based on hospital size, geographic location, and annual ED volume, allowing extrapolation to national estimates. Accordingly, each individual NEISS case corresponds to a larger number of injuries at the national level, and raw case counts should be interpreted in the context of these national sampling weights.

We queried the NEISS for ocular trauma associated with dodgeball, kickball, and pickleball from 2014 to 2023. Because these sports lack individual product codes, we used the broader category of “Other Ball Sports” (Product Code: 3235) for initial identification. Injury narratives were subsequently manually reviewed to confirm explicit documentation of participation in of one of the three sports, as well as ocular and/or orbital injury. Three physician investigators, each board-certified by the American Board of Ophthalmology, independently reviewed all injury narratives to ensure accurate classification and minimize misattribution; discrepancies were resolved by a fourth physician investigator. To capture ocular and periorbital trauma, body part codes for the head (75), face (76), and eye (77) were included in the query.

### Data collection

Cases unrelated to the eye or orbit, unrelated to the specified sports, or with missing or ambiguous data were excluded. For each case, the following variables were collected: age, race, sex, date of injury, location of injury, diagnosis, and ED disposition. Patients were classified into the following age groups: children (0–12), teenagers (13–17), adults (18–65), and elderly (>65). The injury dates from the NEISS were used to assign each case to a season: Spring (March–May), Summer (June–August), Fall (September–November), and Winter (December–February). Diagnoses were determined with NEISS-coded fields and corroborated through narrative review.

Injury mechanisms were categorized into one of ten groups: (1) Hit by Ball in the Eye, (2) Hit by Ball in the Face, (3) Hit by Ball in the Head, (4) Collision with Other Player, (5) Collision with Equipment or Environment, (6) Elbowed in the Eye, (7) Kneed in the Eye, (8) Unknown, (9) Fall, and (10) Foreign Body. Classification uncertainties were resolved through discussion between the physician investigators.

### Statistical analysis

Inter-reviewer agreement for diagnosis, mechanism of injury, and sport assignment was quantified using Fleiss’ kappa across the three independent physician investigators. Descriptive statistics characterized demographic variables, diagnoses, injury mechanisms, and ED dispositions. One-way analysis of variance (ANOVA) assessed differences in mean age between sports. Pearson chi-square tests and odds ratios (ORs) evaluated associations between injury patterns and variables, such as age groups, seasons, injury locations, sex, and race. NEISS weighted-national estimates and data from the U.S. Census Bureau and American Community Survey (ACS) between 2014 to 2023 were used to calculate annual incidence rates per million people. Linear regression was used to examine trends in ocular injury incidence for each sport. Separate linear regression analyses were conducted for NEISS-weighted national injury estimates and for population-adjusted incidence. Additionally, to contextualize pickleball injury trends relative to sport growth, changes in NEISS-weighted national estimates and population-adjusted incidence during the early 2020s were compared with national participation data released by the SFIA.

All statistical analyses were performed through GraphPad Prism (Version 10.4.1 for macOS, GraphPad Software, San Diego, California, USA) and IBM SPSS Statistics (Version 29.0.2.0). *P* values < 0.05 were considered statistically significant. This study was exempt from institutional review board approval by the Albert Einstein College of Medicine, as it used public, de-identified data. The study adhered to the STROBE (Strengthening the Reporting of Observational Studies in Epidemiology) guidelines and was conducted in accordance with the principles of the Declaration of Helsinki.

## Results

### Incidence

**Fig. 1:**
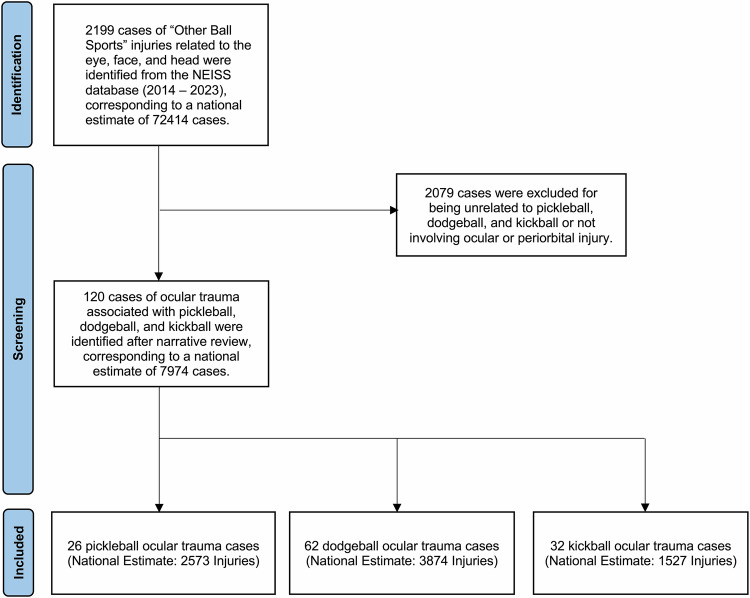
Flow diagram of inclusion criteria for ocular trauma related to pickleball, dodgeball, and kickball from the NEISS database (2014–2023). Flow diagram summarizing the case selection process for ocular injuries associated with dodgeball, kickball, and pickleball using the National Electronic Injury Surveillance System (NEISS). A total of 2199 “Other Ball Sports” cases were identified, corresponding to a national estimate of 72,414 injuries (95% CI: 65,993–78,835). After narrative review and exclusion of non-ocular and unrelated sport injuries, 120 cases, corresponding to 7974 cases nationally (95% CI: 7184–8764), were included: 62 dodgeball (*n* = 3874; 95% CI: 3429–4319), 32 kickball (*n* = 1527, 95% CI: 1255–1799), and 26 pickleball (*n* = 2573, 95% CI: 2218–2928).

Between 2014 and 2023, 2199 cases of ball sports–related trauma were identified in the NEISS, corresponding to a national estimate of 72,414 injuries (95% CI: 65,993–78,835). After applying inclusion criteria and manual review, 120 cases of ocular trauma attributable to dodgeball, kickball, or pickleball were selected, representing a weighted national estimate of 7974 injuries (95% CI: 7184–8764) (Fig. 1). These included 62 dodgeball cases that corresponded to an estimated 3874 injuries (95% CI: 3429–4319), 32 kickball cases to 1527 injuries (95% CI: 1255–1799), and 26 pickleball cases to 2573 injuries (95% CI: 2218–2928). Inter-reviewer reliability for narrative-based case classification was robust (Fleiss’ κ = 0.84), indicating almost perfect agreement among the three independent physician investigators.

**Fig. 2:**
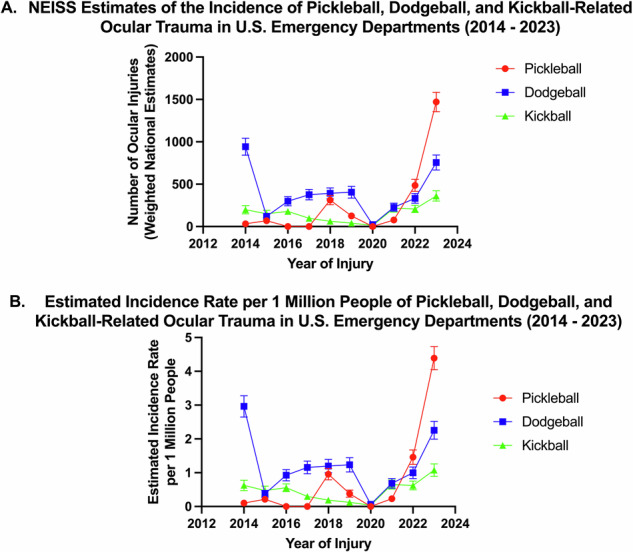
Annual incidence of ocular injuries from pickleball, dodgeball, and kickball in U.S. emergency departments (2014–2023). Panel **A** displays annual NEISS-weighted national estimates of ocular trauma related to dodgeball, kickball, and pickleball, with 95% confidence intervals. Panel **B** presents the corresponding annual incidence rates per million, normalized using population estimates from the United States Census Bureau and American Community Survey. Both panels include 95% confidence intervals. A statistically significant increase was observed in both national estimates and incidence rates for pickleball-related ocular injuries (*P* = 0.04), while trends for dodgeball and kickball remained stable.

Linear regression showed an increase in pickleball-related ocular trauma from 2014 to 2023 using both NEISS-weighted national estimates (Slope: 97.27; R² = 0.42; *P* = 0.04) and incidence per million (Slope: 0.29; R² = 0.42; *P* = 0.04). No trends were observed for dodgeball-related ocular injuries, based on either NEISS-weighted estimates (Slope: –9.76; R² = 0.01; *P* = 0.77) or incidence per million (Slope: –0.04; R² = 0.02; *P* = 0.70). Similarly, kickball-related ocular injuries showed no changes over time using NEISS-weighted estimates (Slope: 10.72; R² = 0.10; *P* = 0.38) or incidence per million (Slope: 0.03; R² = 0.08; *P* = 0.43) (Table 1, Fig. 2). Between 2021 and 2023, NEISS-weighted pickleball ocular injury estimates increased from 77 to 1470 cases (1810% increase), and population-adjusted incidence rose from 0.23 to 4.39 injuries per million (1791% increase).

**Table 1:** Linear regression analysis of annual ocular injury trends for pickleball, dodgeball, and kickball (2014–2023).

Sport of injury	Slope	Standard error	95% Confidence interval	*R*-squared	*P*-Value
Dodgeball (NEISS-weighted estimates)	−9.76	32.05	−83.66–64.15	0.01	0.77
Dodgeball (incidence per million)	−0.04	0.10	−0.27–0.19	0.02	0.70
Kickball (NEISS-weighted estimates)	10.72	11.49	−15.77–37.20	0.10	0.38
Kickball (Incidence per million)	0.03	0.03	−0.05–0.11	0.08	0.43
Pickleball (NEISS-weighted estimates)	97.27	40.45	3.99–190.50	0.42	0.04
Pickleball (incidence per million)	0.29	0.12	0.01–0.57	0.42	0.04

Linear regression was performed on NEISS-weighted national injury estimates and on population-adjusted incidence per million to assess temporal trends in ocular trauma for each sport. The slope, standard error, 95% confidence intervals, coefficient of determination (R²), and *P*-values for trend over time are presented.

Seasonal analysis showed that most pickleball (42.31%) and dodgeball (37.10%) eye injuries occurred in fall, while kickball (37.50%) injuries were most frequently reported in the spring (Table 2). However, Pearson chi-square testing showed that seasonal injury distribution did not differ significantly across sports (*P* = 0.242).

**Table 2:** Comparative epidemiology of pickleball, dodgeball, and kickball eye injuries (2014–2023).

Category	Sub-category	Dodgeball	Kickball	Pickleball
Sex	Males	42 (67.74%)	22 (68.75%)	15 (57.69%)
	Females	20 (32.26%)	10 (31.25%)	11 (42.31%)
Race	White	27 (43.55%)	13 (40.63%)	16 (61.54%)
	Black/African American	8 (12.90%)	10 (31.25%)	2 (7.69%)
	Asian	1 (1.61%)	0	1 (3.85%)
	American Indian/Alaska Native	1 (1.61%)	0	0
	Not Stated	24 (38.71%)	8 (25.00%)	7 (26.92%)
	Other	1 (1.61%)	1 (3.13%)	0
Seasons	Spring	18 (29.03%)	12 (37.50%)	5 (19.23%)
	Summer	7 (11.29%)	8 (25.00%)	6 (23.08%)
	Fall	23 (37.10%)	6 (18.75%)	11 (42.31%)
	Winter	14 (22.58%)	6 (18.75%)	4 (15.38%)
Dispositions	Treated and released	61 (98.39%)	32 (100.00%)	25 (96.15%)
	Treated and admitted for hospitalization	1 (1.61%)	0	0
	Treated and transferred	0	0	1 (3.85%)
Diagnosis	Conjunctivitis	1 (1.61%)	0	0
	Corneal abrasion	20 (32.26%)	5 (15.63%)	7 (26.92%)
	Eye abrasion	1 (1.61%)	0	0
	Eye contusion	9 (14.52%)	3 (9.38%)	1 (3.85%)
	Eye laceration	1 (1.61%)	0	0
	Eyelid abrasion	0	1 (3.13%)	0
	Eyelid contusion	2 (3.23%)	1 (3.13%)	0
	Eyelid ecchymosis	0	1 (3.13%)	0
	Eyelid hematoma	0	1 (3.13%)	0
	Eyelid laceration	2 (3.23%)	4 (12.50%)	1 (3.85%)
	Eyelid swelling	1 (1.61%)	0	1 (3.85%)
	Hyphema	3 (4.84%)	1 (3.13%)	1 (3.85%)
	Iritis	1 (1.61%)	1 (3.13%)	3 (11.54%)
	Orbital contusion	0	1 (3.13%)	0
	Orbital fracture	3 (4.84%)	2 (6.25%)	3 (11.54%)
	Orbital swelling	1 (1.61%)	0	0
	Other	15 (24.19%)	10 (31.25%)	5 (19.23%)
	Subconjunctival hemorrhage	2 (3.23%)	1 (3.13%)	1 (3.85%)
	Uveitis	0	0	1 (3.85%)
	Vitreous detachment	0	0	2 (7.69%)
Mechanism of injury	Hit by ball in the eye	38 (61.29%)	13 (40.63%)	18 (69.23%)
	Hit by ball in the face	13 (20.97%)	5 (15.63%)	0
	Hit by ball in the head	2 (3.23%)	3 (9.38%)	0
	Collision with other player	4 (6.45%)	4 (12.50%)	0
	Collision with equipment or environment	2 (3.23%)	0	2 (7.69%)
	Elbowed in the eye	0	2 (6.25%)	0
	Kneed in the eye	0	1 (3.13%)	0
	Unknown	3 (4.84%)	1 (3.13%)	1 (3.85%)
	Fall	0	2 (6.25%)	4 (15.38%)
	Foreign body	0	1 (3.13%)	1 (3.85%)
Injury locations	School	31 (50.00%)	13 (40.63%)	0
	Sports or recreational center	18 (29.03%)	8 (25.00%)	21 (80.76%)
	Other public property	0	0	1 (3.85%)
	Home	1 (1.61%)	1 (3.13%)	0
	Unknown	12 (19.35%)	10 (31.25%)	4 (15.38%)
Total		62 (100.00%)	32 (100.00%)	26 (100.00%)

Shown are raw case counts for demographics, seasonal patterns, emergency department dispositions, detailed ocular diagnoses, mechanisms of injury, and injury locations. Classification of sport involvement and ocular injury was based on independent manual narrative review.

### Demographics

Across all sports, most injuries occurred in males: 67.74% in dodgeball, 68.75% in kickball, and 57.69% in pickleball. No significant association was found between sex and likelihood of ocular injury by sport (*P* = 0.610).

The age distribution of injured individuals varied significantly by sport (*P* < 0.001). Dodgeball and kickball-related ocular injuries primarily involved children or teenagers, while pickleball-related ocular trauma affected adults and the elderly. One-way ANOVA revealed that the mean age of pickleball ocular trauma patients was 58.27 years (95% CI: 54.64–61.90), significantly higher than that of dodgeball (13.32 years, 95% CI: 10.97–15.67) and kickball ocular trauma patients (14.88 years, 95% CI: 11.60–18.15) (Table 3).

**Table 3:** Comparison of mean age by sport for ocular trauma patients using one-way analysis of variance (ANOVA).

Sport of injury	Mean (years)	Variance	95% CI (years)
Dodgeball	13.32	1.43	10.97–15.67
Kickball	14.88	2.78	11.60–18.15
Pickleball	58.27	3.42	54.64–61.90

One-way ANOVA was used to evaluate differences in the mean age of patients presenting to the emergency department for pickleball and ball sports-related ocular injuries. Mean values, variances, and 95% confidence intervals (CIs) are presented.

White individuals were significantly less likely than non-White individuals to sustain ocular trauma from kickball (OR: 0.33; 95% CI: 0.118–0.921). A trend toward racial differences in the likelihood of ocular trauma was observed, though it did not reach statistical significance (*P* = 0.067).

### Locations, mechanisms of injury, diagnoses, and dispositions

Injury location was significantly different by sport (*P* < 0.001). Most pickleball eye injuries (80.76%) occurred at sports and recreational facilities while dodgeball (50.00%) and kickball (40.63%) injuries primarily occurred at schools (Table 2). Compared to dodgeball and kickball, pickleball ocular injuries were less likely to occur at informal or unsupervised locations such as homes, schools, or general-use public spaces, such as parks (OR: 0.027, 95% CI: 0.003–0.212), and was instead more commonly reported at designated sports or recreational facilities.

Being hit directly by a ball in the eye was the most common mechanism across all three sports: dodgeball (61.29%), kickball (40.63%), and pickleball (69.23%). Falls were more frequently observed as the mechanism of injury while playing pickleball (15.38%) compared to dodgeball and kickball (Table 2).

Corneal abrasions were the most common diagnosis for dodgeball (32.26%), pickleball (26.92%), and kickball (15.63%) ocular trauma. In dodgeball-related cases, other frequent diagnoses included eye contusions (14.52%) and hyphema (4.84%). For kickball, eyelid lacerations (12.50%) and orbital fractures (6.25%) were commonly reported. Pickleball ocular trauma patients also presented with orbital fractures (11.54%), iritis (11.54%), and vitreous detachment (7.69%). Notably, a substantial portion of injuries across all sports were categorized as “Other”: 24.25% in dodgeball, 31.25% in kickball, and 19.23% in pickleball (Table 2).

Most patients were treated and released from the ED: 98.39% in dodgeball, 100.00% in kickball, and 96.15% in pickleball. One patient injured in the eye during dodgeball (1.61%) was admitted for hospitalization, and one patient with pickleball-related ocular trauma (3.85%) was treated and then transferred to another facility. No patients left the ED without being seen (Table 2).

## Discussion

Pickleball-related ocular injuries significantly increased from 2014 to 2023, according to NEISS-weighted estimates (*P* = 0.04), indicating the emergence of pickleball injuries as an increasingly relevant public health concern in ophthalmology. While fewer raw cases were recorded for pickleball than kickball (26 vs 32), the higher national estimates (2573 vs 1527) suggest that pickleball injuries are more commonly captured at large, urban hospitals with greater sampling weight. This reflects pickleball’s increasing popularity among adults, especially in urban and suburban recreational centers typically represented within the NEISS sample. In comparison, ocular injuries from dodgeball and kickball did not change significantly during the study period (Table 1). By presenting both NEISS-weighted national estimates and population-adjusted incidence, our analysis highlights both ED burden and risk in the general population. Despite growth in national pickleball participation, the relative increase in pickleball-related ocular trauma was substantially greater. NEISS-weighted injury estimates increased by 1810%, and population-adjusted incidence increased by 1791%, while national participation in pickleball increased by 158.6% from 2020 to 2023 [7]. Because the number of pickleball cases captured in the NEISS is small, and early-year estimates can fluctuate when case counts are small, these comparisons should be interpreted with caution; however, the discrepancy in magnitude suggests participation growth may not wholly explain an increase in injury rates.

Although pickleball, dodgeball, and kickball represent a small share of the roughly 2.4 million ocular injuries that occur annually in the United States, pickleball’s contribution to annual eye trauma relative to other sports appears to be increasing [10]. Analyses of tennis injuries indicate that ocular trauma in tennis has steadily declined over the past two decades, from approximately 800 cases in the early 2000s to fewer than 500 by 2019 [11]. A similar downward pattern has been observed in baseball, where national estimates decreased to approximately 200 injuries in 2020 [12]. In contrast to declining injury trends in these established sports, the upward trajectory of pickleball-related ocular trauma observed in our analysis suggests that it may represent a growing share of the national burden [13]. Further, in our study, males accounted for the majority of cases, which is consistent with broader epidemiologic patterns in sports-related eye trauma and higher male participation rates in sports across all ages [14]. Additionally, injuries occurred most often in the fall and spring, possibly corresponding with both the academic school year and peak recreational activity among adults (Table 2).

Beyond these trends, age distributions also varied by sport. Pickleball-related ocular trauma disproportionately affected older adults, whereas dodgeball and kickball injuries more frequently occurred among adolescents (Table 3). This age distribution likely reflects differences in participation patterns and risk. Older adults experience age-related declines in reflexes, visual processing, and neuromuscular coordination, reducing their ability to react to fast volleys delivered from distances as short as 14 feet – conditions that require reaction times between 0.35 and 0.40 seconds [8]. These physiological changes, combined with high exposure, may explain the elevated injury burden in this group [15, 16]. However, the risk is not limited to older players. According to data provided by SFIA, youth participation in pickleball is growing fast, with more than 1 million new players under 18 years old between 2022 and 2023 [7]. As additional age groups take up pickleball, the associated risk of ocular trauma may increase among younger athletes.

In terms of injury mechanisms, falls emerged as an important contributor to pickleball-related ocular trauma. Falls accounted for 15.38% of the pickleball-related injuries, possibly reflecting age-related vulnerability, as older adults have a higher baseline risk of falls from impaired balance, coordination, and muscle tone [17]. Visual impairment and loss of contrast sensitivity also contribute to fall risk, particularly in patients with ocular comorbidities, such as age-related macular degeneration (AMD) [18, 19]. In some cases, falls or direct impact led to severe injuries, including orbital fractures. In our analysis, 11.54% of pickleball injuries, 4.84% of dodgeball injuries, and 6.25% of kickball ocular injuries resulted in orbital fractures, warranting closer examination of fracture mechanisms. The hydraulic theory proposes that force applied to the globe rapidly increases intraorbital pressure, fracturing the orbital floor and medial wall, while the buckling theory suggests trauma transmitted through the orbital rim deforms the orbital bones and causes fractures [20]. While our dataset lacks fracture morphology, both models are plausible in the context of pickleball, dodgeball, or kickball-associated ocular trauma, where either globe-directed or rim-mediated forces may act. Among pickleball ocular trauma patients, orbital fractures were associated with both falls and ball strikes, indicating both rim-loading and globe-directed forces may contribute. Ball characteristics, such as mass, velocity, and contact surface area, may influence force transmission to the orbit. Prior studies have shown that sports using small, high-speed projectiles, such as baseball and softball, tend to cause floor and multi-wall fractures consistent with hydraulic injury patterns [21, 22]. These findings imply that sport-specific factors, such as impact dynamics, may influence not only fracture risk but also the biomechanical pathway to injury, highlighting the need for future studies comparing fracture mechanisms across sports.

These fracture patterns also raise important considerations regarding associated neuro-ophthalmic injury. The relationship between orbital fractures and optic nerve trauma remains understudied, as delayed manifestations such as traumatic optic neuropathy can emerge after initial injury. Although optic nerve injury was not reported in our NEISS sample, the observed rate of orbital fractures, especially among older pickleball players, raises concern for neuro-ophthalmic complications. These fractures often occur following falls or high-velocity blunt trauma, both of which can transmit substantial force to the orbit. Cadaver studies suggest that direct impact to the orbital rim can redirect force anteriorly, potentially deflecting it away from the optic canal [23]. This raises the hypothesis that orbital fractures may mitigate posterior segment trauma and optic nerve damage, similar to the protective effect seen with eyewear use [14]. Additionally, medial wall fractures may act as “safety valves”, dissipating intraorbital pressure into the paranasal sinuses and reducing the risk of globe rupture [24]. Clinical studies have reported traumatic optic neuropathy in 3% of orbital blowout fractures and globe rupture in 0.83% of fractures, supporting a potential protective role of certain fracture patterns due to the low incidence of additional severe ocular trauma [25]. In pickleball and similar ball sports, variation in ball size, density, and speed may alter patterns of orbital and globe trauma [26, 27]. Future research should  examine whether specific orbital fracture morphologies correlate with optic nerve preservation, or whether surgical repair impacts force distribution dynamics.

Across all sports, the most common injury mechanism was direct blunt trauma to the globe, either from high-velocity volleys or close-range throws. Injuries of this type can result in eye contusions and hyphema, both established risk factors for angle recession and subsequent traumatic glaucoma [28, 29]. In our study cohorts, eye contusions accounted for 14.52% of dodgeball injuries, 9.38% of kickball injuries, and 3.85% of pickleball injuries, while hyphema accounted for 4.84%, 3.13%, and 3.85%, respectively. Longitudinal follow-up is not available within NEISS, but the presence of such diagnoses suggests a potential for delayed complications and emphasizes the need for vigilance-which is especially compelling when one considers that older adults make up a large percentage of pickleball-related injuries and may have additional age-related ocular vulnerability [30]. Corneal abrasions represented the most common diagnosis across all three sports. Although the majority of corneal abrasions are self-limited and resolve without long-term sequelae, deeper epithelial injuries may result in complications, including ulceration or scarring [31, 32].

Because prior estimates suggest that as many as 90% of sports-related eye injuries can be prevented with proper protective eyewear, a large proportion of pickleball, dodgeball, and kickball injuries are potentially preventable through targeted prevention and sport-specific safety policies [33]. The American Academy of Pediatrics and the American Academy of Ophthalmology have recommended protective eyewear for participants in sports, such as dodgeball, kickball, and pickleball, to reduce ocular trauma risk [34]. Computer-simulated impact studies have demonstrated the ability of polycarbonate and acrylic protective eyewear to absorb and redistribute energy from blunt trauma, decreasing retinal stress by 47–61% and thereby preventing complications, such as retinal detachment or hemorrhage [35]. This protection may result from limiting globe deformation perpendicular to the force of impact, a mechanism implicated in eye contusion injuries from ball sports [36]. Real-world interventions support the use of protective eyewear. In squash players, the Protective Eyewear Promotion (PEP) project combined signage, gear availability, and venue-based outreach, increasing eyewear use by a factor of 2.4 [37]. In women’s lacrosse, a national mandate led to an 84% reduction in eye injuries and a 56% reduction in other head and facial trauma [38]. Although USA Pickleball, the sport’s national governing body, has been called upon to develop formal guidance on protective eyewear, it declined a 2024 proposal requiring eye protection in sanctioned tournaments, citing logistical concerns regarding enforcement.[39].

These findings highlight the importance of tailoring prevention strategies to specific environments. Educational campaigns targeting pickleball players should emphasise the risk of eye trauma and encourage protective eyewear, especially in recreational venues and community centers, where most injuries occurred. Because many clubs and public recreation sites offering pickleball do not require eye protection, increasing both institutional policies and on-site safety practices is an important area for improvement [40]. In schools where dodgeball and kickball are common, similar recommendations can help reduce corneal abrasions, lacerations, contusions, and other ocular injuries. Mandated eye protection in youth sports, such as ice hockey, has proven effective in lowering eye injury risk and long-term complications [41]. As students from non-white communities were overrepresented in our cohort with regard to kickball-related eye injuries, prevention efforts should be coupled with equitable distribution of resources and culturally responsive outreach. Prolonged safety education and better accessibility to protecting devices are required to reduce the burden of eye trauma associated with sports.

While the NEISS provides critical national-level insights, a number of limitations must be considered. First, the NEISS includes only ED presentations, which may underestimate the burden of ocular trauma from these sports because of an exclusion of injuries in outpatient or urgent care settings. NEISS also lacks detailed clinical data, including best-corrected visual acuity, imaging findings, injury severity, and follow-up data. This limits our ability to quantify long-term vision loss or estimate disability-adjusted life years. Because NEISS lacks a validated metric of injury severity, injuries cannot be formally labelled as “serious” or “non-serious.” Nevertheless, the coded diagnoses and narrative descriptions available within NEISS permit descriptive reporting, which is provided in Table 2. This permits the reader to discern comparatively severe injuries, such as orbital fractures or vitreous involvement, versus more minor presentations, such as corneal abrasions. Furthermore, secondary injury data were not systematically reported in NEISS until 2019, thus potentially underestimating associated ocular complications. Also, case identification within the NEISS relies partly on narrative clarity and coding precision. While some residual error remains possible, having three physicians independently review all narratives with adjudication by a fourth reviewer reduced the possibility of misclassification to the greatest extent possible. Finally, though the dataset contained 120 individual cases, each NEISS entry is assigned a sampling weight that reflects a larger number of national injuries. This weighting enhances national representativeness, but subgroup analyses by sport or diagnosis may still be constrained by small raw case counts. Lastly, because NEISS prohibits the release of case-level information that would allow identification, additional appendices of individual injuries with details could not be provided, and all findings can only be reported in aggregate.

In summary, this nationally representative study characterizes the burden of ocular trauma from pickleball, dodgeball, and kickball and reveals distinct variations in age distribution, mechanisms, and diagnoses. Comparing data across sports provides insight into how sport-specific factors drive ocular injury risk. While injuries in dodgeball and kickball largely occurred among youth in school settings, those in pickleball occurred among older adults and were frequently associated with falls and orbital fractures. The growth in pickleball injuries constitutes a new emerging public health concern. These findings support the development of targeted interventions, such as age- and sport-specific protective eyewear, education campaigns, and better injury surveillance. Continued monitoring and prevention efforts will be crucial as participation in athletics continues to increase. By identifying unique risk profiles, this study creates the foundation for efforts to decrease vision loss and enhance safety in both recreational and school-based athletic settings.

## Summary

### What is already known

- Sports-related ocular trauma is a recognized cause of emergency department visits in the United States, with basketball and baseball identified as leading contributors. While prior studies have examined high-profile sports, there is limited national data on eye injuries from recreational ball sports, particularly dodgeball, kickball, and the increasingly popular sport of pickleball. The demographics, mechanisms, clinical outcomes, and injury settings associated with pickleball-related eye trauma have not been comprehensively described using national U.S. population-level data.

### What this study adds

1. This is the first nationally representative study to analyze ocular trauma from dodgeball, kickball, and pickleball using NEISS data from 2014 to 2023, identifying 120 confirmed cases corresponding to a weighted national estimate of 7974 injuries.
2. Pickleball-related ocular injuries are increasing and predominantly affect older adults. These injuries often resulted from direct ball impact or falls and included serious diagnoses such as orbital fractures.
3. Unlike dodgeball and kickball, which occurred primarily in school settings and affected younger individuals, pickleball injuries were more likely to occur in community recreation centers. These findings support the need for age-specific prevention strategies, including education and promotion of protective eyewear for older adults.

## Data Availability

The data supporting this study’s findings are publicly available from the NEISS database (https://www.cpsc.gov/Research--Statistics/NEISS-Injury-Data). Exclusion criteria were applied to the data obtained from NEISS for this study. The data used for this study’s analysis will be available from the authors upon reasonable request.
