# Correction: Pickleball and Ball Sports-Related Ocular Trauma in the United States

**DOI:** 10.1038/s41433-026-04662-3

**Published:** 2026-06-25

**Authors:** Jainam Shah, Sachin Pathuri, Anurag Shrivastava, Joshua Ong, Jason Zheng, Ibrahim Furkan Acar, James Plotnik, Karl Golnik, Mark I. Salevitz, Alex Suh, Andrew G. Lee

**Affiliations:** 1 Albert Einstein College of Medicine, Bronx, NY USA; 2 Department of Ophthalmology and Visual Sciences, Montefiore Medical Center, Albert Einstein College of Medicine, Bronx, NY USA; 3 Department of Ophthalmology and Visual Sciences, University of Michigan Kellogg Eye Center, Ann Arbor, MI USA; 4 California University of Science and Medicine, Colton, CA USA; 5 University of California, Los Angeles, CA USA; 6 Division of Ophthalmology, Phoenix Children’s Hospital, Phoenix, AZ USA; 7 Department of Neurology, Barrow Neurological Institute, Phoenix, AZ USA; 8 Department of Ophthalmology, USC Roski Eye Institute, Keck School of Medicine, University of Southern California, Los Angeles, CA USA; 9 Center for Space Medicine, Baylor College of Medicine, Houston, TX USA; 10 Department of Ophthalmology, Blanton Eye Institute, Houston Methodist Hospital, Houston, TX USA; 11 The Houston Methodist Research Institute, Houston Methodist Hospital, Houston, TX USA; 12 Departments of Ophthalmology, Neurology, and Neurosurgery, Weill Cornell Medicine, New York, NY USA; 13 Department of Ophthalmology, University of Texas Medical Branch, Galveston, TX USA; 14 University of Texas MD Anderson Cancer Center, Houston, TX USA; 15 Texas A&M College of Medicine, Bryan, TX USA; 16 Department of Ophthalmology, The University of Iowa Hospitals and Clinics, Iowa City, IA USA

**Keywords:** Epidemiology, Health care

Correction to: *Eye* 10.1038/s41433-025-04192-4, published online 16 January 2026

The authors would like to correct a minor error noted in the manuscript. Within the “Data collection” subsection of the methods, the numerical age range for teens was incorrectly listed as “(12–17)” and should instead read “(13–17),” as age groups were mutually exclusive in the analyses. This correction does not affect the analyses or results and reflects only a typographical error in the drafting of the methods section. All analyses in the original manuscript used the correct age groupings.

The original article has been corrected.

